# The Impact of Maternal Plant-Based Diet on Obstetric and Neonatal Outcomes—A Cross-Sectional Study

**DOI:** 10.3390/nu15224717

**Published:** 2023-11-08

**Authors:** Paulina Przybysz, Adrian Kruszewski, Joanna Kacperczyk-Bartnik, Ewa Romejko-Wolniewicz

**Affiliations:** 1 Students’ Scientific Group Affiliated to 2nd Department of Obstetrics and Gynecology, Medical University of Warsaw, 00-315 Warsaw, Poland; adrkru7@gmail.com; 2 2nd Department of Obstetrics and Gynecology, Medical University of Warsaw, 00-315 Warsaw, Poland; asiakacperczyk@gmail.com (J.K.-B.); ewamariar@wp.pl (E.R.-W.)

**Keywords:** plant-based diet, pregnancy, gestational diabetes

## Abstract

Background: In the last decades, plant-based diets have gained popularity. Pregnancy is not a contraindication to follow a meat-free diet. This study aimed to compare maternal and neonatal outcomes between women who followed a plant-based diet with those on an omnivore diet. Our second purpose was to investigate the association between physical activity level in combination with diet type and the occurrence of GDM and gestational hypertension. Methods: A questionnaire was distributed electronically via social media. The survey was conducted on a population of Polish women. Results: The final research group included 1015 women. The results showed that a maternal plant-based diet 6 months before pregnancy and during pregnancy does not change the incidence of GDM, anemia, and gestational hypertension. Moreover, no association was found between a diet type before conception and a delivery method or newborn birth weight. Among women who followed an omnivore diet, the risk of GDM was lower in a group with adequate physical activity during 6 months before conception (*p* = 0.0166). However, the combination of a plant-based diet with adequate activity during the preconception period did not influence GDM incidence. Conclusions: Our study indicates that a plant-based diet during the preconception period is not worse than an omnivore diet.

## 1. Introduction

Pregnancy is a unique time. During this period, women’s dietary habits affect the developing fetus. In the last decades, plant-based diets have gained popularity [1]. The pregnancy time and lactation period are not a contraindication to follow a meat-free diet. There are several forms of plant-based diets. The strictest dietary exclusions are followed by vegans, who omit any animal-source food. In Poland in 2014, vegetarians formed 1% of the population, and in 2019, the percentage of people on a vegetarian or vegan diet was 8.4% [2]. The decision to follow a vegetarian diet can be associated with ethical and religious beliefs, lifestyle and health beliefs, and, in some cases, socio-economic constraints. One cause for meat exclusion from a diet may be a growing concern regarding the impact of food production on the environment [3]. 

Plant-based diets have a low content of essential micronutrients such as iron, zinc, vitamin B12, vitamin D, omega-3 (n-3) fatty acids, calcium, and iodine. Microelement deficiencies pose a risk of malnutrition and may lead to adverse effects [4]. On the other hand, the research by Jedut et al. showed that vegetarians have significantly more vitamin A in their diet than omnivores [5]. A plant-based diet is rich in fiber, magnesium, potassium, and antioxidants but presents a lower intake of saturated fatty acids [6]. To date, there are no data that assessed vegetarian eating patterns during pregnancy as nutritionally adequate, but the available literature does not support a negative impact on the mother’s health and pregnancy outcomes [7]. Beneficial aspects of vegetarianism are suggested. Vegetarians have a lower incidence of type 2 diabetes, obesity, ischemic heart disease, and other noncommunicable diseases [8,9]. Meat-free diets are suitable not only for the prevention but also for the treatment of many diseases [10]. The International Agency for Research on Cancer (WHO-IARC) classified the consumption of red meat as “probably carcinogenic to humans” and processed meat as “carcinogenic to humans” [11]. Higher consumption of red meat is associated with a higher risk of diabetes, ischemic heart disease, pneumonia, and diverticular disease. On the other hand, high consumption of red meat reduces the risk of anemia [12].

According to a Academy of Nutrition and Dietetics statement, well-planned vegetarian, including vegan, diets are appropriate for individuals during all stages of the life cycle, including during pregnancy and lactation [10]. In the literature, it is reported that a vegan diet during pregnancy was associated with a reduced risk of maternal excessive weight gain but with adverse effects on a child—increased risk of the small-for-gestational-age and lower-birth-weight centile [13]. The same study showed that vegans have a lower risk of gestational diabetes mellitus (GDM) compared to omnivores, while no such association was observed for vegetarians [13]. A systematic narrative review regarding vegan–vegetarian diets did not find any studies that demonstrated or indirectly suggested a higher risk of severe, adverse pregnancy-related events, such as preeclampsia, HELLP (hemolysis, elevated liver enzymes, low platelet count) syndrome, or major birth defects in women on plant-based diets compared to women with other dietary patterns [14]. Dietary habits influence human milk composition, but, for example, protein concentration does not vary with maternal intake of vegetal or animal proteins [4]. Despite these data, studies shedding light on the possible effect of dietary habits on maternal and infant outcomes are still needed. 

This study aimed to compare maternal and neonatal outcomes in pregnant women on a plant-based diet and those on an omnivore diet. The maternal outcomes were GDM, anemia during pregnancy, gestational hypertension, weight gain during pregnancy, and duration of pregnancy. Neonatal outcomes were newborn birth weight, newborn status at birth, type of delivery, and breastfeeding. This study was also undertaken to assess prepregnancy lifestyle, i.e., how the diet type and the level of physical activity during the 6 months before conception influence the incidence of GDM and gestational hypertension. Also, our purpose was to create a vade mecum about the impact of a preconception plant-based diet on common pregnancy complications for women planning pregnancy and healthcare professionals advising them.

## 2. Materials and Methods

### 2.1. The Cross-Sectional Survey

**Figure 1:**
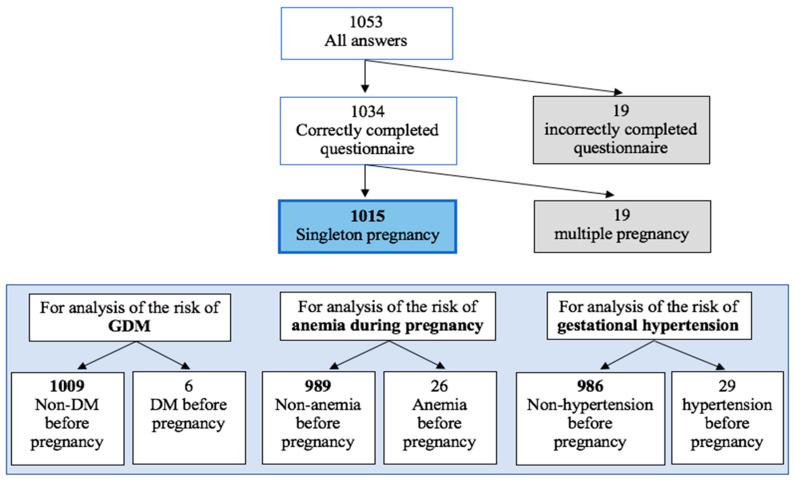
The inclusion and exclusion criteria.

We conducted a cross-sectional survey study. The retrospective analysis involved a sample of Polish women after delivery. The sample size was determined using power analysis. The inclusion criteria were the following: correctly completed questionnaire and singleton delivery. Additionally, for the analysis of the diet type impact on GDM occurrence, participants with diabetes mellitus (type I or type II) diagnosed before pregnancy were excluded. Analogously, women diagnosed with anemia before pregnancy were excluded from the analysis of the diet type impact on the risk of anemia during pregnancy, and women diagnosed with hypertension before pregnancy were excluded from the analysis of gestational hypertension risk (Figure 1).

### 2.2. The Statistical Analysis and Endpoints

The analysis of the diet type influence was performed separately for the diet followed during 6 months before pregnancy and for the diet followed during pregnancy. We also analyzed the percentage of women who changed their diet type after conception. 

The endpoints included the development of GDM, anemia in pregnancy and gestational hypertension, weight gain during pregnancy, pregnancy duration, and type of delivery. For the analysis of the GDM and gestational hypertension risk, we also added, as a confounding factor, the impact of physical activity during the 6 months before conception. An additional maternal endpoint was effective breastfeeding from the first day of the newborn’s life. In the analysis of the association between maternal diet and the newborn condition, the endpoints were the newborn’s birth weight and 5th minute Apgar score result. 

The survey was self-composed by the authors. The anonymous questionnaire consisted of single-choice, multiple-choice closed, and open questions. The survey was voluntary, and it was distributed electronically using Google Forms (Google LLC, Mountain View, CA, USA). The full version of the survey is available in the Supplementary Materials. 

### 2.3. The Study Group

The questionnaire was distributed via randomly chosen Polish maternity and parental Facebook groups. The data were collected between November 2022 and January 2023. The survey was conducted among 1057 women. The final study group included 1015 women. Of the 1057 women, 6 women had pregestational diabetes mellitus (type I or type II), 26 women had anemia before pregnancy, and 29 women had chronic hypertension or were diagnosed with hypertension before the 20th week of pregnancy. These women were excluded from the corresponding analyses, respectively, as described above.

Respondents were divided into two groups: women on a “plant-based diet” (on a vegetarian or vegan diet during the 6 months before pregnancy) and women on an “omnivore diet” (on a diet containing meat meals during the 6 months before pregnancy).

During pregnancy, anemia is defined as a hemoglobin concentration (Hb) < 110 g/L at sea level [15]. Gestational hypertension according to the American College of Obstetricians and Gynecologists guidelines is defined as blood pressure greater than or equal to 140 mmHg systolic or 90 mmHg diastolic after the 20th week of pregnancy when previous blood pressure was normal [16]. Less physically active women were defined as those who spent less than 90 min a week on physical activity and adequately physically active women were those who spent more than 90 min on exercise a week [17].

### 2.4. The Endpoint Definitions

Prepregnancy body mass index (BMI) was calculated by dividing prepregnancy weight in kilograms by height in meters squared. According to the World Health Organization criteria, the following ranges were defined: underweight: <18.5 kg/m^2^; normal weight: 18.5–24.9 kg/m^2^; overweight: 25–29.9 kg/m^2^; and obese: ≥30.0 kg/m^2^. According to the prepregnancy BMI, the gestational weight gain in kilograms was estimated using the recommendations of the Institute of Medicine and The National Research Council of the National Academies (US) [18]. The women were assigned to one of the following categories: too little weight gain, adequate weight gain, and too much weight gain. 

Delivery before 37 completed weeks of gestation was considered as premature birth and delivery after 42 completed weeks of gestation was defined as post-term delivery. Delivery between the 37th and 41st week of gestation was considered as term delivery. The delivery methods were divided into vaginal deliveries (natural, forceps, vacuum extractor), cesarean sections performed due to the medical indications, and cesarean sections performed due to undefined or unclear indications. The presence or absence of the cesarean section indication was determined by the answer analysis to the open question about the reason for the cesarean section and compared with the national recommendations of the Polish Society of Gynecologists and Obstetricians [19]. Newborn birth weight was divided into three ranges: below 2500 g, between 2500 g and 4000 g, and above 4000 g.

Statistical analysis of the responses to the survey was performed in STATISTICA 13.3 (TIBCO Software Inc., Palo Alto, CA, USA). The groups of “plant-based diet” and “omnivore diet” were compared. The questionnaire data were analyzed using Fisher exact tests and power analysis. Statistically significant *p*-values were considered less than 0.05. Chi-square analysis and logistic regression analysis was performed in PQStat Software (1.8.6.102 version). The impact of the additional factors (BMI) was calculated using log-linear analysis (a version of chi-square analysis to examine more than two categorical variables).

## 3. Results

### 3.1. Study Population

The research group consisted of 1015 women after delivery. A total of 40.9% of surveyed women had delivered in 2022, 24.6% of women had delivered in 2021, 11.0% in 2020 and 23.4% had delivered before 2020. The characteristics of the division according to diet type during the 6 months before conception are presented in Table 1. Statistically significant differences between the subgroups concerned the place of residence and the prepregnancy BMI. A total of 6.0% of vegetarians and vegans lived in a small town of up to 50,000 inhabitants compared to 15.9% of women on an omnivore diet (*p* = 0.0153). Omnivores were overweight more than twice as often as vegetarians and vegans (19.2% vs. 8.4%; *p* = 0.0119). 

### 3.2. Diet Type

Only 8.2% (N = 83) of respondents were on a plant-based diet during the 6 months before pregnancy, vegetarians constituted 5.7% (N = 58), and vegans 2.5% (N = 25) of the total study group. A total of 14.5% (N = 12) of them switched to an omnivore diet after getting pregnant. Only 1.7% (N = 16) of women who ate meat before conception decided to refrain from eating meat during pregnancy. Pregnancy was the reason why the group of vegetarians and vegans significantly more often than omnivores decided to change their eating habits (*p* < 0.00001). A total of 19% of vegetarian women changed their habits to an omnivore diet after conception. Only 4% of vegans switched to an omnivore diet, and 8% of vegans switched to a vegetarian diet after conception. The post hoc calculated power of this analysis was 99.1%.

### 3.3. Gestational Diabetes Mellitus (GDM)

The association between the diet type and GDM incidence is presented in Table 2. No association was found between dietary habits before conception and the risk of developing GDM, but the type of diet during pregnancy was associated with GDM occurrence. It was observed in 8.1% of women on a plant-based diet and in 15.5% on an omnivore diet. Multivariate analysis revealed an influence of prepregnancy BMI on the relationship between diet type and GDM incidence—the GDM risk increased with the prepregnancy BMI increase in the omnivore group.

The impact of diet type combined with physical activity level during the prepregnancy period on GDM occurrence is presented in Table 3. The history of adequate physical activity before pregnancy was not a protective factor against developing GDM among women on a plant-based diet. In women on a meat-containing diet, GDM was diagnosed in 16.6% of those who were less physically active and only in 9.6% of those who were adequately physically active (*p* = 0.0166). This analysis has a 68.7% post hoc calculated power.

### 3.4. Anemia during Pregnancy

The diet type impact on anemia occurrence during pregnancy is presented in Table 4. Almost every third woman following a plant-based diet and every fourth woman following an omnivorous diet before conception developed anemia during pregnancy. However, this difference was not found to be statistically significant—neither in a univariate nor in a multivariate analysis. Among women following a plant-based diet during pregnancy, 27.7% had anemia, and among omnivorous women during pregnancy—24.6%. The type of diet during pregnancy was not associated with the occurrence of anemia. Multivariate analysis showed that prepregnancy BMI did not interfere with the relationship between diet type and anemia in pregnancy.

### 3.5. Hypertension in Pregnancy

A total of 3.6% of women who declared vegetarianism or veganism before conception had hypertension diagnosed for the first time during pregnancy, while this complication concerned 6.5% of respondents declaring a diet including meat meals. This relationship was not statistically significant in univariate analysis (*p* = 0.47). However, a multivariate analysis indicated that prepregnancy BMI is a contributory factor influencing the association between the diet type before pregnancy and the incidence of gestational hypertension. The higher the prepregnancy BMI, the higher the gestational hypertension risk. The type of diet during pregnancy was not associated with the presence of gestational hypertension (*p* = 0.07). This complication was observed in 2.3% of women on a plant-based diet and 6.7% of women on an omnivore diet. Multivariate analysis showed that prepregnancy BMI was a contributing factor to gestational hypertension incidence in the plant-based diet group, though the numbers are small; see Table 5. 

The impact of diet type combined with physical activity level 6 months before conception on the incidence of gestational hypertension is presented in Table 6. No association was found between the type of diet and level of activity before conception on the occurrence of gestational hypertension.

### 3.6. Weight Gain during Pregnancy

Insufficient weight gain was recorded in 34.9% of respondents who before conception followed a plant-based diet, and in 32.1% of women on the omnivore diet (*p* = 0.62). Too much weight gain presented in 27.7% of vegetarians and vegans compared to 34.3% of women on an omnivore diet; the difference was not statistically significant. The type of diet before conception did not affect weight gain during pregnancy. Multivariate analysis revealed an influence of prepregnancy BMI on the relationship between the diet type and gestational weight gain; overweight and obese women on an omnivore diet gained too much weight more frequently, and similarly, overweight women presented insufficient weight gain on an omnivore diet (Table 7).

### 3.7. Duration of Pregnancy

Preterm delivery occurred with similar frequency in both subgroups. There were no statistically significant differences between subgroups in the frequency of post-term deliveries. No association was found between the diet type during the 6 months before conception and the pregnancy duration in univariate analysis, but multivariate analysis revealed that prepregnancy BMI was a contributory factor influencing the pregnancy duration in diet type subgroups, i.e., overweight and obese women on an omnivore diet more frequently delivered prematurely and post-term (Table 7).

### 3.8. Newborn Birth Weight

The percentage of newborns with low birth weight (<2500 g) was similar in both subgroups. Similarly, the subgroups did not differ in offspring’s birth weight above 4000 g. No association between diet type during the 6 months before conception and newborn birth weight was observed, and multivariate analysis showed no impact of prepregnancy BMI on this relationship (Table 7).

### 3.9. Newborn Status at Birth

We divided newborns into two subgroups: those with an Apgar score higher than or equal to 8 points and those with a score less than 8 points. Children of mothers on a plant-based diet did not differ significantly in Apgar scores from children of mothers on a diet including meat meals in univariate analysis. Multivariate analysis showed that women with higher prepregnancy BMI on an omnivore diet delivered children in worse condition (Apgar score less than 8 points) (Table 7).

### 3.10. Type of Delivery

The results regarding the prepregnancy diet impact on the type of delivery are presented in Table 8. Vegetarians and vegans had fewer cesarean sections due to medical indications than omnivores (32.5% vs. 42.7%), but it was not statistically significant. The frequency of vaginal delivery was not associated with the type of diet. Dietary habits during the 6 months before conception did not impact the type of delivery in univariate analysis. Newborn birth weight was the main factor contributing to a delivery method: children below 2500 g were more often delivered by cesarean section in the plant-based diet group. 

### 3.11. Breastfeeding

A total of 897 respondents declared that they breastfed their child, which was 88.4% of our total study group. Among the breastfeeding women, we analyzed the association between diet during the 6 months before pregnancy and the occurrence of breastfeeding difficulties (Table 9). A total of 24.7% of women on a plant-based diet reported breastfeeding difficulties, while among omnivores, this percentage was 33.7% (*p* = 0.13). Dietary habits in the preconception period were not associated with breastfeeding difficulties.

### 3.12. Summary

The performed logistic regression analyses showed that in terms of gestational diabetes mellitus, anemia during pregnancy, and gestational hypertension, a plant-based diet in the preconception period does not differ from an omnivorous diet. Analysis showed no correlation between the type of diet and presence of anemia during pregnancy (OR < 1). A summary of the obtained results is presented in Table 10.

## 4. Discussion

In this study, a plant-based diet was relatively common among pregnant women, with 5.7% of them being vegetarian and 2.5% being fully vegan based on reported dietary habits. The results showed that a plant-based diet—both during the preconception period and during pregnancy—was not associated with the incidence of anemia during pregnancy and gestational hypertension in univariate analysis. Multivariate analysis showed that prepregnancy BMI was a contributing factor to gestational hypertension incidence in the plant-based diet group, though the numbers are small. Additionally, a meat-free diet during the preconception period did not change the risk of developing GDM but during pregnancy was associated with a lower incidence of GDM. The GDM risk increased with the prepregnancy BMI increase in the omnivore group. There were fewer overweight women in the plant-based group than in the omnivore group, and the incidence of GDM was also affected by prepregnancy BMI, as shown by multivariate analysis.

The mechanism of plant-based-diet action on organisms is various. This diet has a beneficial effect on the microbiome by inducing the development of more diverse and stable microbial systems, hence supporting overall health [20]. The literature indicates that vegetarianism can increase nitric oxide bioavailability and decrease reactive oxygen species [6]. The semiquantitative review conducted by Jaworsky et al. of women with high risk for GDM pointed out that a plant-based diet and phytochemicals may reduce blood glucose and improve antioxidant activity to reduce the oxidative stress that is often associated with GDM [21]. Also, plant-rich meals significantly increase the serum levels of glucagon-like peptide-1 (GLP-1), a hormone that augments the secretion of insulin [6]. In our paper, we focused on the impact of diet on chosen maternal and neonatal outcomes, but further studies examining the impact and its mechanisms are needed.

The research by Yisahak et al. included 1948 low-risk pregnant women of four races in the USA, and the results showed that vegetarianism did not exhibit associations with maternal outcomes including GDM, hypertensive disorders, and anemia [22]. There is evidence that a higher intake of red and processed meat is a risk factor for developing GDM [23] and type 2 diabetes mellitus [24,25].

The study conducted by Carter et al. revealed that a vegan diet (low in arachidonic acid) might provide protection against preeclampsia and could alleviate most if not all of the signs and symptoms of preeclampsia [26]. Research performed by Pistollato et al. suggests that maternal nutritional patterns characterized by a low intake of plant-derived foods could increase the risk of gestation-related issues, such as preeclampsia and pregravid obesity, and contribute to the onset of pediatric diseases [27]. 

The result of our research showed that physical activity over 90 min a week is associated with a lower incidence of GDM. However, increasing physical activity among women on a plant-based diet does not affect the risk of GDM. On the other hand, among women on an omnivore diet, physical activity is associated with a lower incidence of GDM. We did not observe that the type of diet and increased physical activity were additional factors reducing the risk of gestational hypertension. The risk of gestational hypertension is strongly related to BMI, as mentioned above. The study conducted by Kruszewski A, Przybysz P., et al., which included over 960 women, showed that GDM is more common in women who did not exercise at all or had physical activity less than 90 min a week during the 6 months before pregnancy [17].

The previous studies on the impact of maternal diet on a newborn’s birth weight were limited and yielded inconsistent findings. Piccoli et al. in a systematic narrative review reported that five studies found lower birth weight in neonates of vegetarian mothers, whereas two analyses suggested higher birth weight [14]. Our findings showed no association between maternal diet and newborn birth weight. The percentage of newborns with too low body weight (<2500 g) was similar in both subgroups. Moreover, neonates of women on a plant-based diet had similar Apgar score results at the 5th minute after birth compared to neonates of omnivorous women. Newborns who received 8 or more points in the Apgar score constituted 98.8% and 96.6% of the above-mentioned groups, respectively. Moreover, multivariate analysis showed that women with higher prepregnancy BMI on an omnivore diet delivered children in worse condition (Apgar score less than 8 points). We did not observe an association between the type of delivery (vaginal or cesarean section) and maternal diet. In a multivariate analysis, a relationship was found between the newborn birth weight and the type of delivery.

According to a paper by Kesary et al., a maternal vegetarian diet was associated with a lower risk of excessive weight gain, and there was no statistical evidence for an association between maternal diet and preterm delivery or low birth weight [13]. We did not observe an increased risk of preterm birth in the group of women on a plant-based diet compared to omnivorous women in univariate analysis, but multivariate analysis revealed that prepregnancy BMI was a contributory factor influencing the pregnancy duration in diet type subgroups, i.e., overweight and obese women on an omnivore diet more frequently delivered prematurely and post-term. The study by Yisahak et al. also showed that diet-based full vegetarians had marginally increased odds of inadequate gestational weight gain during the second trimester [22]. Our univariate analysis of the Polish population showed that the type of diet 6 months before conception did not impact weight gain during pregnancy. Multivariate analysis revealed an influence of prepregnancy BMI on the relationship between diet type and gestational weight gain: overweight and obese women on an omnivore diet gained too much weight more frequently, and similarly, overweight women presented insufficient weight gain on an omnivore diet.

The questionnaire used in this study was self-composed by the authors and it had not been previously validated in a pregnant population. The limitation was that we could not verify all the answers. Some answers were subjective and self-reported by participants. The limitation of our survey was the fact that women could only choose the way of eating in closed questions distinguishing between a meat and a meatless diet, and women did not specify what type of food they consumed. We cannot rule out that a confounding bias and a misclassification bias may have occurred because of this. Unfortunately, we have no information on whether both types of diet were well-balanced and provided all the necessary micronutrients. An additional limitation was no information about the caloric content of the diet.

In a study by Marangoni F. et al., it was observed that even in the most industrialized countries, specific dietary intakes during pregnancy and lactation are often inadequate. This particularly applies to DHA, calcium, iron, folic acid, iodine, and vitamin D [28]. This result coincides with research on the Polish population that showed that none of the studied women managed adequate nutrition in terms of all tested macro- and micronutrients [29]. Particular attention should be paid to women of childbearing age following exclusion diets such as veganism because of the increased risk of inadequate supply of nutrients. Findings in the meta-analysis by Bhutta et al. confirm that supplementation during pregnancy reduces low birth weight in the population at risk [30]. Conclusions from the paper by Avnon et al. are that a vegan diet does not change the umbilical cord levels of B12, folic acid, ferritin, and hemoglobin. Moreover, at greater risk of B12 deficiency are vegans who do not take any vitamin supplementation compared with omnivores [31].

The WHO recommends initiation of breastfeeding within 1 h after birth, exclusive breastfeeding of infants till 6 months of age, and continued breastfeeding until 2 years of age or older [32]. Our research showed that a maternal plant-based diet before conception and during pregnancy is not associated with the occurrence of breastfeeding difficulties.

Our analysis showed that, despite the abundant evidence stating that a plant-based diet during pregnancy is safe for both the mother and the fetus, many women refrain from continuing this diet after conception. As many as one in seven women (14.5%) on a meat-free diet switched to an omnivore diet after becoming pregnant. It is worth considering why women adjust their dietary habits. Our study showed that only 36.8% of surveyed women received advice about nutrition during pregnancy from a healthcare professional such as a doctor, midwife, or dietician. Lack of adequate dietary counseling could be the reason for preference for extensive dietary changes during pregnancy, i.e., reintroducing meat after being vegan or vegetarian.

Our study stands out in that we examined nine associations (in heterogeneous groups, due to exclusions, Figure 1, Materials and Methods) between the type of diet that women follow during the preconception period and the incidence of pregnancy-complicating diseases, premature birth, type of delivery, and neonatal parameters such as birth weight and Apgar score results. Furthermore, we also evaluated the association between diet and breastfeeding. Overweight women statistically significantly more often were on omnivore than plant-based diets, and this difference between subgroups could be the same limitation in our study. Another limitation could be the fact that the number of women on a plant-based diet is relatively small compared to the number of women in the omnivore group.

This study aimed to create a scientific paper that would be a compendium of knowledge for women planning pregnancy about the impact and safety of a properly balanced plant-based diet during pregnancy. Our findings are in line with the statements of the American Dietetic Association that well-planned vegetarian diets are appropriate during all stages of the life cycle including pregnancy [33]. We expect an increase in the population on a plant-based diet. In the coming years, the number of diet-related diseases will change.

## 5. Conclusions

A properly balanced plant-based diet during the preconception period and pregnancy do not change the risk of pregnancy complications such as GDM, anemia, and gestational hypertension. The findings prove that the offspring of women on a plant-based diet are not at higher risk of prematurity or low birth weight. Furthermore, women on a plant-based diet during pregnancy do not experience more problems with breastfeeding than women on an omnivore diet. The factor that influenced the majority of obstetrics complications was prepregnancy BMI. A varied and balanced meat-free diet can be followed by women during pregnancy. 

## Figures and Tables

**Table 1:** Characteristics of the study group and subgroups according to the diet followed during the 6-month period before conception.

	Total Study Group N = 1015	Plant-Based Diet Subgroup N = 83	Omnivore Diet Subgroup N = 932	*p*
	% (N)	% (N)	% (N)	
Age (years)				
16–20	1.9 (19)	1.2 (1)	1.9 (18)	1.00
21–25	12.3 (125)	6.0 (5)	12.9 (120)	0.08
26–30	36.9 (375)	44.6 (37)	36.3 (338)	0.15
31–35	33.2 (337)	28.9 (24)	33.6 (313)	0.47
≥36	15.7 (159)	19.3 (16)	15.3 (143)	0.36
Habitation				
Countryside	19.4 (197)	16.9 (14)	19.7 (183)	0.66
Town < 50,000	15.1 (153)	6.0 (5)	15.9 (148)	0.0153
City 50,000–100,000	10.4 (106)	14.5 (12)	10.1 (94)	0.26
City 100,000–500,000	14.0 (142)	16.9 (14)	13.7 (128)	0.41
City > 500,000	41.1 (417)	45.8 (38)	40.7 (379)	0.42
Education				
Primary	0.6 (6)	0.0 (0)	0.6 (6)	1.00
Vocational	2.2 (22)	2.4 (2)	2.1 (20)	0.70
Secondary	22.2 (225)	19.3 (16)	22.4 (209)	0.58
Higher	75.1 (762)	78.3 (65)	74.8 (697)	0.51
Prepregnancy BMI (kg/m^2^)				
Underweight (<18.5)	7.7 (78)	12.0 (10)	7.3 (68)	0.13
Normal (18.5–24.9)	66.0 (670)	75.9 (63)	65.1 (607)	0.05
Overweight (25–29.9)	18.3 (186)	8.4 (7)	19.2 (179)	0.0119
Obese (≥30)	8.0 (81)	3.6 (3)	8.4 (78)	0.14

**Table 2:** Incidence of GDM according to the diet type during the 6 months before conception and during pregnancy.

Prepregnancy BMI	Plant-Based Diet Subgroup	Omnivore Diet Subgroup		
	% (N)	% (N)	*p* ^a^	*p* ^b^
During the 6 months before pregnancy				
Gestational Diabetes Mellitus (GDM)				
Underweight	11.1 (1)	5.7 (8)	0.26	0.0002
Normal	66.7 (6)	52.5 (74)		
Overweight	22.2 (2)	25.5 (36)		
Obese	0.0 (0)	16.3 (23)		
Nongestational Diabetes Mellitus (Non-GDM)				
Underweight	12.2 (9)	7.6 (60)		
Normal	77.0 (57)	67.8 (532)		
Overweight	6.8 (5)	17.7 (139)		
Obese	4.0 (3)	6.9 (54)		
		
During pregnancy				
Gestational Diabetes Mellitus (GDM)				
Underweight	14.3 (1)	5.6 (8)	0.0495	0.0001
Normal	71.4 (5)	52.4 (75)		
Overweight	14.3 (1)	25.9 (37)		
Obese	0.0 (0)	16.1 (23)		
Nongestational Diabetes Mellitus (Non-GDM)				
Underweight	11.4 (9)	7.7 (60)		
Normal	75.9 (60)	67.8 (529)		
Overweight	8.9 (7)	17.6 (137)		
Obese	3.8 (3)	6.9 (54)		

*p* ^a^—*p* value regarding the relationship between the type of diet and the incidence of GDM; *p* ^b^—*p* value regarding the relationship between the type of diet, GDM, and BMI.

**Table 3:** Presence of GDM according to the diet type and level of physical activity during the 6 months before conception.

	Subgroup	Less Physically Active	Adequately Physically Active	*p*
	% (N)	% (N)	% (N)	
	Plant-based diet			
Non-GDM	89.2 (74)	91.7 (44)	85.7 (30)	0.48
GDM	10.8 (9)	8.3 (4)	14.3 (5)	
	Omnivore diet			
Non-GDM	84.8 (785)	83.4 (616)	90.4 (169)	0.0166
GDM	15.2 (141)	16.6 (123)	9.6 (18)	

**Table 4:** Presence of anemia during pregnancy according to the diet type during the 6month period before conception and during pregnancy.

Prepregnancy BMI	Plant-Based Diet Subgroup	Omnivore Diet Subgroup		
	% (N)	% (N)	*p* ^a^	*p* ^b^
During 6 months before pregnancy				
Anemia				
Underweight	16.7 (4)	8.4 (19)	0.24	0.05
Normal	70.8 (17)	69.9 (158)		
Overweight	8.3 (2)	15.5 (35)		
Obese	4.2 (1)	6.2 (14)		
Nonanemia				
Underweight	10.9 (6)	7.0 (49)		
Normal	76.4 (42)	63.5 (446)		
Overweight	9.1 (5)	20.4 (143)		
Obese	3.6 (2)	9.1 (64)		
During pregnancy				
Anemia				
Underweight	17.4 (4)	8.4 (19)	0.53	0.08
Normal	65.2 (15)	70.5 (160)		
Overweight	13.0 (3)	15.0 (34)		
Obese	4.3 (1)	6.2 (14)		
Nonanemia				
Underweight	10.0 (6)	7.0 (49)		
Normal	76.7 (46)	63.4 (442)		
Overweight	10.0 (6)	20.4 (142)		
Obese	3.3 (2)	9.2 (64)		

*p* ^a^—*p* value regarding the relationship between the type of diet and anemia; *p* ^b^—*p* value regarding the relationship between the type of diet, anemia, and BMI.

**Table 5:** Presence of hypertension in pregnancy according to the diet type during the 6 months before conception and during pregnancy.

Prepregnancy BMI	Plant-Based Diet Subgroup	Omnivore Diet Subgroup		
	% (N)	% (N)	*p* ^a^	*p* ^b^
During 6 months before pregnancy				
Gestational Hypertension				
Underweight	0.0 (0)	3.4 (2)	0.26	<0.0001
Normal	33.3 (1)	45.8 (27)		
Overweight	33.3 (1)	32.2 (19)		
Obese	33.3 (1)	18.6 (11)		
Nonhypertension				
Underweight	12.5 (10)	7.7 (65)		
Normal	77.5 (62)	67.3 (568)		
Overweight	7.5 (6)	18.4 (155)		
Obese	2.5 (2)	6.6 (56)		
During pregnancy				
Gestational Hypertension				
Underweight	0.0 (0)	3.3 (2)	0.07	0.0001
Normal	50.0 (1)	45.0 (27)		
Overweight	50.0 (1)	31.7 (19)		
Obese	0.0 (0)	20.0 (12)		
Nonhypertension				
Underweight	11.7 (10)	7.7 (65)		
Normal	75.3 (64)	67.5 (566)		
Overweight	9.4 (8)	18.2 (153)		
Obese	3.5 (3)	6.6 (55)		

*p* ^a^—*p* value regarding the relationship between the type of diet and gestational hypertension; *p* ^b^—*p* value regarding the relationship between the type of diet, gestational hypertension, and BMI.

**Table 6:** Presence of gestational hypertension according to the diet type and level of physical activity during the 6 months before conception.

	Subgroup	Less Physically Active	Adequately Physically Active	*p*
	% (N)	% (N)	% (N)	
	Plant-based diet subgroup			
Gestational hypertension	3.6 (3)	4.2 (2)	2.9 (1)	1.00
Nonhypertension	96.4 (80)	95.8 (46)	97.1 (34)	
	Omnivore diet subgroup			
Gestational hypertension	6.5 (59)	5.8 (42)	9.3 (17)	0.09
Nonhypertension	93.5 (844)	94.2 (679)	90.7 (165)	

**Table 7:** Weight gain during pregnancy, pregnancy duration, newborn birth weight, and Apgar score at 5 min after birth according to diet type during the 6 months before conception.

Prepregnancy BMI	Plant-Based Diet Subgroup	Omnivore Diet Subgroup		
	% (N)	% (N)	*p* ^a^	*p* ^b^
Weight gain during pregnancy				
Insufficient weight gain				
Underweight	10.3 (3)	8.7 (26)	0.463	<0.001
Normal	86.2 (25)	75.6 (226)		
Overweight	3.4 (1)	15.7 (47)		
Obese	0.0 (0)	0.0 (0)		
Adequate weight gain				
Underweight	19.4 (6)	7.0 (22)		
Normal	67.7 (21)	69.0 (216)		
Overweight	6.5 (2)	14.4 (45)		
Obese	6.5 (2)	9.6 (30)		
Excessive weight gain				
Underweight	4.3 (1)	6.3 (20)		
Normal	73.9 (17)	51.6 (165)		
Overweight	17.4 (4)	27.2 (87)		
Obese	4.3 (1)	15.0 (48)		
Pregnancy duration				
Premature birth				
Underweight	12.5 (1)	8.8 (9)	0.23	0.04
Normal	75.0 (6)	60.8 (62)		
Overweight	12.5 (1)	17.6 (18)		
Obese	0.0 (0)	12.7 (13)		
	Term delivery			
Underweight	14.3 (9)	7.0 (44)		
Normal	71.4 (45)	65.3 (411)		
Overweight	9.5 (6)	19.1 (120)		
Obese	4.8 (3)	8.6 (54)		
Post-term delivery				
Underweight	0.0 (0)	7.5 (15)		
Normal	100.0 (12)	66.7 (134)		
Overweight	0.0 (0)	20.4 (41)		
Obese	0.0 (0)	5.5 (11)		
Newborn birth weight				
Birth weight < 2500 g				
Underweight	20.0 (1)	14.8 (8)	0.16	0.06
Normal	60.0 (3)	63.0 (34)		
Overweight	20.0 (1)	16.7 (9)		
Obese	0.0 (0)	5.6 (3)		
Birth weight 2500–4000 g				
Underweight	12.2 (9)	6.9 (55)		
Normal	77.0 (57)	66.0 (523)		
Overweight	8.1 (6)	18.7 (148)		
Obese	2.7 (2)	8.4 (67)		
Birth weight > 4000 g				
Underweight	0.0 (0)	6.7 (7)		
Normal	75.0 (3)	60.6 (63)		
Overweight	0.0 (0)	24.0 (25)		
Obese	25.0 (1)	8.7 (9)		
Apgar score at 5 min after birth				
≥ 8				
Underweight	12.2 (10)	7.6 (68)	0.21	0.001
Normal	75.6 (62)	65.6 (590)		
Overweight	8.5 (7)	19.2 (173)		
Obese	3.7 (3)	7.7 (69)		
<8				
Underweight	0.0 (0)	0.0 (0)		
Normal	100.0 (1)	53.1 (17)		
Overweight	0.0 (0)	18.8 (6)		
Obese	0.0 (0)	28.1 (9)		

*p* ^a^—*p* value regarding univariate analysis; *p* ^b^—*p* value regarding multivariate analysis.

**Table 8:** Method of delivery according to diet type during the 6 months before pregnancy.

Newborn Birth Weight	Plant-Based Diet Subgroup	Omnivore Diet Subgroup		
	% (N)	% (N)	*p* ^a^	*p* ^b^
Type of delivery				
Vaginal delivery				
<2500 g	1.9 (1)	3.3 (17)	0.16	0.036
2500 g–4000 g	92.5 (49)	86.0 (443)		
>4000 g	5.7 (3)	10.7 (55)		
Cesarean section (medical indications)				
<2500 g	14.8 (4)	5.8 (23)		
2500 g–4000 g	81.5 (22)	81.9 (326)		
>4000 g	3.7 (1)	12.3 (49)		
Cesarean section (undefined indications)				
<2500 g	0.0 (0)	5.3 (1)		
2500 g–4000 g	100.0 (3)	94.7 (18)		
>4000 g	0.0 (0)	0.0 (0)		

*p* ^a^—*p* value regarding the relationship between the type of diet and type of delivery; *p* ^b^—*p* value regarding the relationship between the type of diet, type of delivery, and newborn birth weight.

**Table 9:** The occurrence of breastfeeding difficulties according to diet type during the 6 months before pregnancy.

	Plant-Based Diet Subgroup	Omnivore Diet Subgroup	*p* ^1^
	% (N)	% (N)	
Nonbreastfeeding difficulties	75.3 (58)	66.3 (544)	0.13
Breastfeeding difficulties	24.7 (19)	33.7 (276)	

*p* ^1^—*p* value regarding the relationship between the type of diet and breastfeeding difficulty.

**Table 10:** The presence of differences between women on a plant-based diet and on an omnivorous diet (univariate regression analysis, 95% CI).

Outcome	Plant-Based or Omnivorous Diet 6 Months before Pregnancy			
	Odds Ratio	95% CI	*p*	AUC
Gestational diabetes mellitus	1.134	0.790–1.628	0.494	0.596 ± 0.0262
Anemia during pregnancy	0.877	0.681–1.129	0.308	0.544 ± 0.0209
Gestational hypertension	1.217	0.669–2.213	0.521	0.663 ± 0.0368
	**Plant-based or Omnivorous Diet during Pregnancy**			
Gestational diabetes mellitus	1.350	0.905–2.014	0.141	0.602 ± 0.0260
Anemia during pregnancy	0.939	0.729–1.210	0.625	0.539 ± 0.0208
Gestational hypertension	1.580	0.770–3.240	0.212	0.666 ± 0.0366

CI—confidence interval, AUC—area under the ROC curve.

## Data Availability

All data generated or analyzed during this study are included in this published article and its Supplementary Materials.

## Supplementary Materials

The following supporting information can be downloaded at: https://www.mdpi.com/article/10.3390/nu15224717/s1, File S1: The questionnaire.

## References

[1] Alcorta A., Porta A., Tárrega A., Alvarez M.D., Vaquero M.P. (2021). Foods for Plant-Based Diets: Challenges and Innovations. Foods.

[2] Economic Weekly—Polish Economic Institute. https://pie.net.pl/wp-content/uploads/2021/11/Tygodnik-Gospodarczy-PIE_44-2021.pdf.

[3] Segovia-Siapco G., Sabaté J. (2019). Health and sustainability outcomes of vegetarian dietary patterns: A revisit of the EPIC-Oxford and the Adventist Health Study-2 cohorts. Eur. J. Clin. Nutr..

[4] Sebastiani G., Herranz Barbero A., Borrás-Novell C., Alsina Casanova M., Aldecoa-Bilbao V., Andreu-Fernández V., Pascual Tutusaus M., Ferrero Martínez S., Gómez Roig M.D., García-Algar O. (2019). The Effects of Vegetarian and Vegan Diet during Pregnancy on the Health of Mothers and Offspring. Nutrients.

[5] Jedut P., Glibowski P., Skrzypek M. (2023). Comparison of the Health Status of Vegetarians and Omnivores Based on Biochemical Blood Tests, Body Composition Analysis and Quality of Nutrition. Nutrients.

[6] Schiattarella A., Lombardo M., Morlando M., Rizzo G. (2021). The Impact of a Plant-Based Diet on Gestational Diabetes: A Review. Antioxidants.

[7] Baroni L., Rizzo G., Goggi S., Giampieri F., Battino M. (2021). Vegetarian diets during pregnancy: Effects on the mother’s health. A systematic review. Food Funct..

[8] Key T.J., Papier K., Tong T.Y.N. (2022). Plant-based diets and long-term health: Findings from the EPIC-Oxford study. Proc. Nutr. Soc..

[9] Le L.T., Sabaté J. (2014). Beyond meatless, the health effects of vegan diets: Findings from the Adventist cohorts. Nutrients.

[10] Melina V., Craig W., Levin S. (2016). Position of the Academy of Nutrition and Dietetics: Vegetarian Diets. J. Acad. Nutr. Diet..

[11] Bouvard V., Loomis D., Guyton K.Z., Grosse Y., Ghissassi F.E., Benbrahim-Tallaa L., Guha N., Mattock H., Straif K., International Agency for Research on Cancer Monograph Working Group (2015). Carcinogenicity of consumption of red and processed meat. Lancet Oncol..

[12] Papier K., Fensom G.K., Knuppel A., Appleby P.N., Tong T.Y.N., Schmidt J.A., Travis R.C., Key T.J., Perez-Cornago A. (2021). Meat consumption and risk of 25 common conditions: Outcome-wide analyses in 475,000 men and women in the UK Biobank study. BMC Med..

[13] Kesary Y., Avital K., Hiersch L. (2020). Maternal plant-based diet during gestation and pregnancy outcomes. Arch. Gynecol. Obstet..

[14] Piccoli G.B., Clari R., Vigotti F.N., Leone F., Attini R., Cabiddu G., Mauro G., Castelluccia N., Colombi N., Capizzi I. (2015). Vegan-vegetarian diets in pregnancy: Danger or panacea? A systematic narrative review. BJOG.

[15] World Health Organization (2001). Iron Deficiency Anemia Assessment Prevention and Control: A Guide for Programme Managers.

[16] (2020). Gestational Hypertension and Preeclampsia, ACOG Practice Bulletin, Clinical Management Guidelines for Obstetrician-Gynecologists, Number 222. Obstet. Gynecol..

[17] Kruszewski A., Przybysz P., Kacperczyk-Bartnik J., Dobrowolska-Redo A., Romejko-Wolniewicz E. (2023). Physical Activity during Preconception Impacts Some Maternal Outcomes—A Cross-Sectional Study on a Population of Polish Women. Int. J. Environ. Res. Public Health.

[18] IOM (2009). Weight Gain during Pregnancy: Reexamining the Guidelines.

[19] Wielgoś M., Bomba-Opoń D., Bręborowicz G.H., Czajkowski K., Dębski R., Leszczyńska-Gorzelak B., Oszukowski P., Radowicki S., Zimmer M. (2018). Recommendations of the Polish Society of Gynecologists and Obstetricians regarding caesarean sections. Ginekol. Pol..

[20] Moszak M., Szulińska M., Bogdański P. (2020). You Are What You Eat-The Relationship between Diet, Microbiota, and Metabolic Disorders-A Review. Nutrients.

[21] Jaworsky K., DeVillez P., Basu A. (2023). The Role of Phytochemicals and Plant-Based Diets in Gestational Diabetes: Evidence from Clinical Trials. Int. J. Environ. Res. Public Health.

[22] Yisahak S.F., Hinkle S.N., Mumford S.L., Li M., Andriessen V.C., Grantz K.L., Zhang C., Grewal J. (2021). Vegetarian diets during pregnancy, and maternal and neonatal outcomes. Int. J. Epidemiol..

[23] Bao W., Bowers K., Tobias D.K., Hu F.D., Zhang C. (2013). Prepregnancy dietary protein intake, major dietary protein sources, and the risk of gestational diabetes mellitus: A prospective cohort study. Diabetes Care.

[24] The InterAct Consortium (2013). Association between dietary meat consumption and incident type 2 diabetes: The EPICInterAct study. Diabetologia.

[25] Schwingshackl L., Hoffmann G., Knu S. (2017). Food groups and risk of type 2 diabetes mellitus: A systematic review and metaanalysis of prospective studies. Eur. J. Epidemiol..

[26] Carter J.P., Furman T., Hutcheson H.R. (1987). Preeclampsia and reproductive performance in a community of vegans. South Med. J..

[27] Pistollato F., Sumalla Cano S., Elio I., Masias Vergara M., Giampieri F., Battino M. (2015). Plant-Plant-Based and Plant-Rich Diet Patterns during Gestation: Beneficial Effects and Possible Shortcomings. Adv. Nutr..

[28] Marangoni F., Cetin I., Verduci E., Canzone G., Giovannini M., Scollo P., Corsello G., Poli A. (2016). Maternal Diet and Nutrient Requirements in Pregnancy and Breastfeeding. An Italian Consensus Document. Nutrients.

[29] Mierzejewska E. (2022). Comprehensive Assessment of pro-Health Behaviors and Their Determinants among Pregnant Women in the Context of Current National and International Recommendations on the Management of Healthy Pregnancy. Unpublished. Doctoral Dissertation.

[30] Bhutta Z.A., Das J.K., Rizvi A., Gaffey M.F., Walker N., Horton S., Webb P., Lattey A., Black R.E., Lancet Nutrition Interventions Review Group (2013). Evidence-based interventions for improvement of maternal and child nutrition: What can be done and at what cost?. Lancet.

[31] Avnon T., Anbar R., Lavie I., Ben-Mayor Bashi T., Paz Dubinsky E., Shaham S., Yogev Y. (2020). Does vegan diet influence umbilical cord vitamin B12, folate, and ferritin levels?. Arch. Gynecol. Obstet..

[32] Dyson L., McCormick F.M., Renfrew M.J. (2005). Interventions for promoting the initiation of breastfeeding. Cochrane Database Syst. Rev..

[33] Craig W.J., Mangels A.R. (2009). Position of the American Dietetic Association: Vegetarian diets. J. Am. Diet Assoc..

