# Supplementary material for: The Impact of Maternal Plant-Based Diet on Obstetric and Neonatal Outcomes—A Cross-Sectional Study
Source: Nutrients. 2023 Nov 8;15(22):4717. doi: 10.3390/nu15224717 (PMC10675710; doi:10.3390/nu15224717)
Supplement: Supplementary file 1 [file nutrients-15-04717-s001.zip › nutrients-2652758-supplementary.pdf]

The impact of maternal plant-based diet on obstetrics and neonatal outcomes - A cross-sectional study

Supplementary Materials:

Supplement 1. The questionnaire

1. How old were you during your last pregnancy?
2. Where do you live?
    a. in the countryside
    b. in a town < 50.000 inhabitants
    c. in a city 50.000 – 100.000 inhabitants
    d. in a city 100.000 – 500.000 inhabitants
    e. in a city > 500.000 inhabitants
3. What is the level of your education?
    a. primary
    b. vocational
    c. secondary
    d. higher
4. How tall in centimeters are you?
5. How much did you weigh before your last pregnancy?
6. How many kilograms did you gain during the last pregnancy?
7. Was your last pregnancy singleton or multiple?
    a. singleton pregnancy
    b. multiple pregnancy
8. What year did you give birth to your last child?
    a. 2022
    b. 2021
    c. 2020
    d. before 2020
9. What were your dietary habits during the 6 months before your last pregnancy?
    a. a diet containing meat meals
    b. vegetarian diet
    c. vegan diet
10. What were your dietary habits during your last pregnancy?
    a. a diet containing meat meals
    b. vegetarian diet
    c. vegan diet
11. Please mark which sentence best describes your physical activity during the 6 months before last pregnancy.
    a. I did not exercise before pregnancy.
    b. Before pregnancy, my physical activity level was moderate, i.e. I exercised less than 90 minutes a week.
    c. I was physically active before pregnancy, i.e. I exercised more than 90 minutes a week.
12. Please mark which sentence best describes your physical activity during last pregnancy.
    a. I did not exercise during pregnancy, even though I had no medical contraindications
    b. I did not exercise during pregnancy because I had medical contraindications
    c. during pregnancy, my physical activity level was moderate, i.e. less than 90 minutes of physical activity per week

    d. I was physically active during pregnancy; my activity level was more than 90 minutes a week.

13. Please mark which sentence best describes your mental well-being before last pregnancy.
    a. I had no mental disorder
    b. I have experienced serious problems with low mood, but I have not been diagnosed with depression.
    c. I was suffering from depression.
14. Did you receive advice during your last pregnancy from a qualified healthcare professional (doctor /midwife/dietician) regarding pregnancy nutrition?
    a. yes
    b. no
15. Did you receive advice during your last pregnancy from a qualified healthcare professional (doctor /midwife) regarding vitamin and mineral supplementation?
    a. yes
    b. no
16. Which of the nutrients did you take as supplementation during pregnancy? (multiple choice question)
    a. Folic acid
    b. DHA - (polyunsaturated fatty acids)
    c. Iron
    d. Iodine
    e. Vitamin D
    f. Magnesium
    g. Vitamin B12
    h. Other - what?
    i. I did not take supplements
17. Have you experienced hyperemesis gravidarum (severe, persistent vomiting, often associated with weight loss)?
    a. yes
    b. no
18. Have you had gestational diabetes mellitus?
    a. yes
    b. no
    c. I had diabetes mellitus before pregnancy
19. Were you diagnosed with anemia during last pregnancy (anemia/hemoglobin less than 11 g/dL in the first and third trimesters and less than 10.5 g/dL in the second trimester)?
    a. yes
    b. no
    c. I had anemia before the pregnancy
20. Please mark which sentence describes your last pregnancy
    a. I have not had a pregnancy complicated by high blood pressure
    b. I was diagnosed with hypertension before pregnancy
    c. I was diagnosed with hypertension for the first time during pregnancy
21. (If in 20 - C. I was diagnosed with hypertension during pregnancy) In which week of pregnancy were you diagnosed with high blood pressure?
22. (If in 20 – B or C) Please mark which sentence describes your pregnancy.
    a. I have not experienced complications of high blood pressure
    b. I have been diagnosed with pre-eclampsia
    c. I have been diagnosed with eclampsia.

23. What was the week of your last pregnancy at delivery?
24. How did you deliver your last child?
    a. natural birth
    b. forceps delivery/delivery using a vacuum extractor
    c. caesarean section
25. If you selected a cesarean section in the previous question, please state the reason for the caesarean section. (open question)
26. What was the newborn birth weight?
    a. < 2500 g
    b. 2501-3250 g
    c. 3251-4000 g
    d. > 4000 g
27. What was the newborn's birth length?
28. How many points on the Apgar scale did your child receive at the 5th minute of life?
29. After delivery, was your baby referred to the Neonatal Pathology Unit and/or the Neonatal Intensive Care Unit?
30. Were you able to breastfeed immediately after delivery?
31. Did you breastfeed your baby?
    a. yes
    b. no
32. (If in 31 - Yes) Have you experienced problems related to the lack of breast milk?
    a. yes
    b. no
33. (If in 31 - no) If you did not breastfeed, please give the reason. (open question)
34. Please mark which sentence describes how you felt after giving birth.
    a. I haven't experienced any mental disorder.
    b. I experienced the so-called "baby blues" (temporary mood depression, appearing 2-5 days after delivery)
    c. I experienced postpartum depression (symptoms of depression - lack of joy with the baby, including depressed mood, decreased appetite, lack of energy, and sleep problems - occurring within 6 weeks after delivery).
